# Exposure of Children to Rural Lifestyle Factors Associated With Protection Against Allergies Induces an Anti-Neu5Gc Antibody Response

**DOI:** 10.3389/fimmu.2019.01628

**Published:** 2019-07-16

**Authors:** Remo Frei, Caroline Roduit, Ruth Ferstl, Liam O'Mahony, Roger P. Lauener

**Affiliations:** ^1^Christine Kuehne–Center for Allergy Research and Education (CK-CARE), Davos, Switzerland; ^2^Swiss Institute of Allergy and Asthma Research (SIAF), University of Zurich, Zurich, Switzerland; ^3^Children's Hospital, University of Zurich, Zurich, Switzerland; ^4^Children's Hospital of Eastern Switzerland, St. Gallen, Switzerland; ^5^Departments of Medicine and Microbiology, APC Microbiome Ireland, University College Cork, Cork, Ireland

**Keywords:** N-glycolylneuraminic acid, asthma, regulatory T cells, anti-inflammatory, animal contact, hygiene hypothesis

## Abstract

Rural lifestyle has been shown to be highly protective against the development of allergies. Contact to farm-animals or pets and early-life consumption of milk products turned out to be important. These exposures provide contact to N-glycolylneuraminic acid (Neu5Gc), a sialic acid naturally expressed in mammalians but not in humans or microbes although both are able to incorporate exogenously provided Neu5Gc and induce thereby an anti-Neu5Gc antibody response. Farmers' children had elevated levels of anti-Neu5Gc antibodies associated with increased contact to Neu5Gc. Farm-related exposures that were associated with protection against allergies such as exposure to farm-animals or pets and consumption of milk were also associated with an antibody response to Neu5Gc in children. Exposure to cats was associated with increased anit-Neu5Gc IgG levels at different timepoints assessed between 1 year of age and school-age. Moreover, consumption of non-pasteurized milk in the first year of life was associated with increased anti-Neu5Gc IgG levels. Neu5Gc-providing exposures that were associated with protection against allergies were reflected in an elevated anti-Neu5Gc IgG level in children. Exposure to Neu5Gc was associated with anti-inflammation and protection of asthma development in children and mice without contribution of anti-Neu5Gc antibodies.

The term “hygiene hypothesis” has emerged to describe the correlation between the increase in hygienic conditions and the elevation in allergic disorders and asthma, but also in autoimmune and inflammatory diseases in westernized countries over the last decades (1). The hygiene hypothesis was proposed after a lower risk of hay fever and atopic sensitization among children having more siblings was observed (2). The protective effect was assigned to an altered exposure to microbes during childhood. Other findings supported this hypothesis: early entry to day care of the children had a protective effect against the development of allergies and Italian military students with antibodies to hepatitis A virus had a lower prevalence of atopy and atopic respiratory diseases (3, 4). More recent studies have consistently linked altered exposures to microbes early in life with later life risk of allergy and asthma (5). Further, rural lifestyle, especially farm environment, turned out to be highly protective (6–11). We found that, in addition to microbial exposure, rural lifestyle also provide increased exposure to non–microbial-derived N-glycolylneuraminic acid (Neu5Gc) which was reflected by increased levels of anti-Neu5Gc antibodies (12). Although detailed underpinning immunological mechanisms were not fully known, this was the first study showing an immune-modulatory, anti-inflammatory effect of Neu5Gc itself, which is in contrast to findings of xenograft rejection and tumor growth where anti-Neu5Gc antibodies were associated with a pro-inflammatory status (13–19).

Neu5Gc is a specific marker for non-human cells and proteins. Neu5Gc is a sialic acid composed of nine carbon sugars found at the most outer unit of the cellular glycocalyx and on secreted glycoproteins in most mammalian cells. In contrast to all other mammals, including primates, humans and new world monkeys lack the enzyme CMP-Neu5Ac hydroxylase (CMAH) due to a mutation in the respective gene and are therefore not able to synthesize Neu5Gc from the precursor N-acetylneuraminic acid (Neu5Ac) (20–22). Humans are able to uptake Neu5Gc via fluid pinocytosis and a specific lysosomal transporter and incorporate it in newly synthesized glycoproteins. Known dietary sources of Neu5Gc are red meat and cow's milk products (23–25). As a consequence, humans mount a humoral immune response upon exposure to other mammalian cells by producing anti-Neu5Gc immunoglobulins (26, 27).

Rural lifestyle factors described to be protective against the development of allergies and asthma are contact to more and more-diverse microbes in the environment, contact to farm-animals or pets, and altered nutrition such as consumption of more milk products (9, 11, 28–31). To investigate which of the protective farm-exposures is associated with induction anti-Neu5Gc antibodies, we measured anti-Neu5Gc IgG in sera of children from a random sample of the cross-sectional PARSIFAL study (Prevention of Allergy Risk factors for Sensitization In children related to Farming and Anthroposophic Lifestyle) (*n* = 299) including school-age children and from the longitudinal PASTURE study (Protection against Allergy-Study in Rural Environments) including serum samples from cord blood (*n* = 836), 1 year (*n* = 734), 4.5 years (*n* = 700), and 6 years (*n* = 728). Anti-Neu5Gc IgG levels were assessed by quantifying anti-Neu5Gc-polyacrylamid IgG corrected by subtraction of anti-Neu5Ac-polyaccrylamid IgG, which is certainly an underestimation of the anti-Neu5Gc response (23, 26, 32). Anti-Neu5Gc IgG levels were associated with rural environmental exposures reflecting protection against allergies and asthma (contact to farm-animals assessed by the presence in facilities related to livestock, such as stables and contact to pets; nutritional factor such as consumption of cow's milk; contact to microbes assessed by levels of endotoxin and extracellular polysaccharide in children's mattresses) (Table 1).

**Table 1:** Association between farm-related exposures and anti-Neu5Gc IgG levels.

	**Longitudinal^§^**				**Cross-sectional^°^**
**GMR [95% CI]**	**Cord blood**	**1 year**	**4.5 years**	**6 years**	**School-age**
**Contact to animal-related exposure**					
Stable contact ^1^	0.77 [0.25, 2.36]	0.78 [0.15, 4.02]	5.54 [1.34, 22.92]^*^	1.26 [0.39, 4.14]	3.79[1.7, 8.45]^***^
Contact to pets:^1^					
Cats	1.59 [0.65, 3.91]	14.61 [2.06, 103.68]^**^	3.72 [1.34, 10.37]^*^	5.50 [2.13, 14.22]^***^	1.71 [1.14, 2.57]^**^
Dogs	2.23 [0.93, 5.37]	2.37 [0.24, 23.84]	1.13 [0.40, 3.19]	0.70 [026, 1.85]	1.57 [0.88, 2.8]
**Nutrition**					
Non-pasteurized vs. pasteurized milk consumption ^1^	0.88 [0.32, 2.40]	23.92 [5.13, 111.57]^***^	1.60 [0.52, 4.97]	1.49 [0.52, 4.3]	1.43 [0.9, 2.27]
Cow's milk vs. no-milk consumption ^1^		5.57 [1.67, 18.58]^**^	0.50 [0.15, 1.73]	0.63 [0.17, 2.34]	
**Microbal exposure**					
Endotoxin					1.12 [0.95, 1.31]
Extracellular polysaccharide					1.11 [0.97, 1.27]

§ *GMR (geometric mean ratio), adjusted for center, farmer, parental atopy, sex, and duration of breastfeeding*.

1 *Cord blood: associations with exposure during pregnancy; 1 year, 4.5 years, and 6 years: associations with exposure during the last 12 months; for exposure during the first year of life, adjustment for prenatal exposure*.

° *GMR. Variables: adjusted for farming, sex, and age*.

*Bold values were statistically significant*.

*Two-sided P < 0.05 were considered significant*.

* *P < 0.05*;

** *P < 0.01*;

*** *P < 0.001*.

Contact to animals specially contact to cats was strongly and consistently associated with increased anti-Neu5Gc IgG antibodies. Contact to cats was associated with elevated antibody levels at different timepoints assessed between 1 year of age and school-age, while contact to a stable environment was significantly associated with anti-Neu5Gc IgG levels in the school-age children of the cross-sectional study and in 4.5 years old children of the longitudinal study (Table 1). Worth mentioning is that children having contact to cats had a reduced risk to develop asthma later in life [adjusted odds ratio (aOR) for exposure to cat during pregnancy and asthma at 6 years: 0.6 (95% confidence interval (CI) 0.36–1); aOR for exposure to cat in the first year of life and asthma at 6 years: 0.59 (95% CI 0.35–0.98); aOR for exposure to cat at 4 years of age and asthma at 6 years: 0.52 (95%CI 0.31–0.87)]. Moreover, cow's milk consumption was significantly associated with increased anti-Neu5Gc IgG levels at 1 year of age. Remarkable, consumption of non-pasteurized milk was much stronger associate with anti-Neu5Gc antibody titers compared to pasteurized milk consumption, although we don't know if pasteurization reduces the immunogenicity of Neu5Gc bearing proteins (Table 1). Finally, exposure to microbial components such as endotoxin or extracellular polysaccharide in mattresses of the school-age children was not significantly associated with anti-Neu5Gc IgG levels.

The data outlined above show that exposure to rural lifestyle factors, which provide protection against the development of allergies or asthma and additionally provide contact to Neu5Gc such as contact to cats or early-life consumption of farm-milk induced an antibody response against Neu5Gc. Contact to microbes, not expressing Neu5Gc was not associated with anti-Neu5Gc levels. Elevated anti-Neu5Gc IgG levels were associated with dietary Neu5Gc intake and infections with certain viruses such as Epstein-Barr virus (13, 33). Our data indicate that also inhaled Neu5Gc provided by contact to cats induced an anti-Neu5Gc antibody response, although it is still possible that inhaled antigens reach the intestine.

The route of initial antigen exposure is crucial in determining whether tolerance or allergic sensitization occurs (34). In contrast to early-life exposure of antigens via the skin, which has been proposed as a key route for allergic sensitization to allergens, exposure via the oral route is associated with induction of tolerance. Inhaled antigens reaching the intestine or orally ingested antigens are encountered by the intestinal-draining lymph nodes which is also the site of antigen-specific regulatory T cell generation a crucial event for tolerance induction (34). Induction of tolerance is one key mechanism how environmental factors might protect children from the development of allergies and asthma (28, 30, 35). We have recently shown that Neu5Gc-feeding of mice had protective effects in models of airway and gut inflammation in the absence of anti-Neu5Gc antibodies (12). The beneficial effect of Neu5Gc was based on anti-inflammatory effects such as increased numbers of regulatory T cells and elevated expression of indoleamine 2,3-dioxygenase (IDO), retinaldehyde dehydrogenase 2 (RALDH2), and IL-10 in dendritic cells and on a reduction of T helper cell type 17 responses (12). Moreover, Massoud et al. showed that intravenous immunoglobulin therapy was able to attenuate airway hyperresponsiveness and inflammation mediated by regulatory T cells in a model of airway inflammation in mice. This effect was dependent on intravenous immunoglobulin sialylation, because neuraminidase-treated intravenous immunoglobulin was not able to induce regulatory T cells and intravenous immunoglobulin enriched in sialic acid displayed an enhanced anti-inflammatory activity. Furthermore, the authors showed that the anti-inflammatory effect of sialylated intravenous immunoglobulin was mediated by binding to the C-type lectin dendritic cell immunoreceptor (DCIR) (36, 37).

Other mechanisms underpinning the protective effect of Neu5Gc contact might be mediated by Neu5Gc binding to Sialic acid-binding Ig-like lectins (Siglecs). Siglecs have an intracellular immunoreceptor tyrosine-based inhibitory motifs (ITIM) thereby inhibiting activation signals by activation of Src homology region 2 domain-containing phosphatase (SHP)-1 and−2, tyrosine phosphatase, and suppressor of cytokine signaling (SOCS)-3 (38–40). Especially, activation of Siglec-8 on eosinophils by sialic acids turned out to be a promising target for asthma treatment because of induction of apoptosis of those cells (41). These data indicate that exposure to Neu5Gc might support tolerance development.

We suggest that Neu5Gc behaves as an anti-inflammatory molecule in the human immune system that is able to prevent the development of asthma symptoms but also colitis. The loss of Neu5Gc during human evolution, possibly driven by selective pressure of a pathogen, may have removed this anti-inflammatory molecule and consequently removed one of the “brakes” previously used to limit immune pathology (42). Exposure to animals or animal-derived foods containing Neu5Gc seems to replace this molecule and assist immune regulatory processes. However, the move away from traditional farming environments limits the exposure to Neu5Gc and thereby might contribute to exaggerated inflammatory responses. Future studies should consider the deliberate exposure to Neu5Gc as a novel anti-inflammatory strategy but also involve studies using Neu5Gc together with anti-Neu5Gc antibodies since there are studies showing a pro-inflammatory role of Neu5Gc in combination with anti-Neu5Gc antibodies in tumor and xenograft models (13–19).

## Ethics Statement

This study was carried out in accordance with the recommendations of guidelines of the ethics committee of the canton St. Gallen with written informed consent from all subjects. All subjects gave written informed consent in accordance with the Declaration of Helsinki. The protocol was approved by the ethics committee of the canton St. Gallen.

## Author Contributions

RFr, CR, RFe, LO, and RL conceived and designed the experiments, discussed the data, and wrote the paper.

### Conflict of Interest Statement

The authors declare that the research was conducted in the absence of any commercial or financial relationships that could be construed as a potential conflict of interest.
